# Analyzing the Compressive Strength of Ceramic Waste-Based Concrete Using Experiment and Artificial Neural Network (ANN) Approach

**DOI:** 10.3390/ma14164518

**Published:** 2021-08-11

**Authors:** Hongwei Song, Ayaz Ahmad, Krzysztof Adam Ostrowski, Marta Dudek

**Affiliations:** 1 College of Civil Engineering, Dalian Minzu University, Dalian 116650, China; shwdut2000@163.com; 2 Department of Civil Engineering, Abbottabad Campus, COMSATS University Islamabad, Abbottabad 22060, Pakistan; 3 Faculty of Civil Engineering, Cracow University of Technology, 24 Warszawska Str., 31-155 Cracow, Poland; marta.dudek@pk.edu.pl

**Keywords:** ceramic waste powder, concrete, cement, artificial neural network, prediction, machine learning algorithms

## Abstract

In a fast-growing population of the world and regarding meeting consumer’s requirements, solid waste landfills will continue receiving a substantial amount of waste. The utilization of solid waste materials in concrete has gained the attention of the researchers. Ceramic waste powder (CWP) is considered to be one of the most harmful wastes for the environment, which may cause water, soil, and air pollution. The aim of this study was comprised of two phases. Phase one was based on the characterization of CWP with respect to its composition, material testing (coarse aggregate, fine aggregate, cement,) and evaluation of concrete properties both in fresh and hardened states (slump, 28 days compressive strength, and dry density). Concrete mixes were prepared in order to evaluate the compressive strength (CS) of the control mix, with partial replacement of the cement with CWP of 10 and 20% by mass of cement and 60 prepared mixes. However, phase two was based on the application of the artificial neural network (ANN) and decision tree (DT) approaches, which were used to predict the CS of concrete. The linear coefficient correlation (R^2^) value from the ANN model indicates better performance of the model. Moreover, the statistical check and k-fold cross validation methods were also applied for the performance confirmation of the model. The mean absolute error (MAE), mean square error (MSE), and root mean square error (RMSE) were evaluated to confirm the model’s precision.

## 1. Introduction

One of the challenging tasks for the world is to utilize the waste materials obtained from various industries, such as ceramic waste powder (CWP) [1]. It has been reported that 1.4 million tons of ceramic waste are being produced from ceramic manufacturing per year in the EU alone, and the amount of ceramic waste produced in Europe from different production stages of the ceramic industry is increasing and the majority of this waste is being disposed of in landfills. [2]. The strict rules in the European union regarding landfills have resulted in the increase of the cost for its deposition. Industries will have to take an alternative solution for the reuse of ceramic waste. The practical application of this waste has been practiced in numerous industrial sectors with its limited usage [3,4]. However, the construction industry plays a vital role to reuse all such types of waste to minimize the environmental risks [5,6,7]. Environmental conditions are severely affected by waste obtained from industries, which alternately plays a role in the increase of global warming [8]. This threat to the environment can be minimized by reprocessing the waste materials to produce effective and environmentally friendly materials, like concrete, which can accommodate many waste materials by replacing the cement in it at a certain amount [9,10,11,12].

Concrete is one of the most famous and high-demand materials all over the world [13]. The use of cement as a binder material in concrete also increases as the demand of concrete increases [14]. A huge number of gases are generated during the manufacturing of cement, and about 4 billion tons of Portland cement (PC) is generated per annum and around one ton of PC produces one ton of CO_2_ [15]. This indicates not only the environmental risks but also increases the global warming [16]. The process of cement replacement with waste materials are playing a positive role in reducing the risks [17]. The different metallurgical wastes such as steel slag, basic oxygen furnace slag (BOFS), red mud, and other wastes are also widely used for cement replacement in concrete [18,19]. Early attempts of incorporating the ceramic powder in concrete was not effective due to inexperience and poor type of powder, but it shows better performance later when proper engineering practices were adopted with the application of various types of ceramic powder in concrete [20]. The replacement of this waste in concrete not only showed a satisfactory performance of concrete for use in construction projects, but also minimizes the environmental risks [21,22]. The application of waste material in concrete not only improves its mechanical properties, but also makes it durable and fulfills the increasing demand of the concrete [23,24,25].

The application of various waste material in concrete not only showed satisfactory results for concrete’s properties, but also made a positive impression on environmental conditions. Siddique et al. [26] conducted a study on the sustainable utilization of ceramic waste in concrete, in which they use different percentages of waste to analyze the properties of concrete. The waste was used as a coarse aggregate to produce durable concrete. El-Dieb et al. [27] studied CWP as an alternative replacement of cement in concrete in terms of characterization and evaluation. They put forward the idea that CWP can be used as an alternative ingredient, which can partially replace the cement and can improve the durability of concrete. Sarkar et al. [28] studied the application of partial replacement of metakaolin with ceramic waste in a geopolymer. They observed mortar with 33 and 50% ceramic waste gives the maximum compressive strength. Xu et al. [29] study was based on the use of ceramic waste tile powder to investigate the properties of low-carbon ultra-high-performance concrete incorporating its various percentages in concrete.

The most important parameter of concrete is its compressive strength (CS), which cannot be ignored in any type of construction work [30]. To obtain the desired compressive strength, it usually undergoes a time-consuming process using the hit and trial method for 28 days. However, supervised machine learning (ML) algorithms are of great interest in the field of civil engineering to predict the strength properties of concrete. The ML approaches generally use various input variables to run the model for the predicted outcome at a certain accuracy. Abuodeh et al. [31] uses deep ML techniques to forecast and assess ultra-high-performance concrete, and an analytical model was developed for this forecasting purpose. They used the proposed artificial neural network (ANN) for the prediction in which the correlation coefficient R^2^ value was about 80%, indicating a high accuracy level of the model. Feng et al. [32] represents the application of an adaptive boosting approach for the prediction of concrete compressive strength. They employed 1030 datasets to train the model and achieved an accuracy of 98%. DeRousseau et al. [33] worked on the various ML methods for comparison in predicting the compressive strength of field-placed concrete. The random forest approach was applied and obtained an R^2^ value of 0.51. Ahmadi et al. [34] conducted work on the application of the ANN approach for predicting the compressive strength of circular steel confined concrete, in which he represents the performance of the ANN model for 268 datasets. Lee et al. [35] present the performance of ANN for the prediction of concrete strength and they recommended the ANN algorithms for accurate prediction of the compressive strength of concrete. Basyigit et al. [36] present their study on the prediction of compressive strength of heavyweight concrete using the ANN technique, in which they use 45 experimental results for running the models. Nguyen et al. [37] present the compressive strength of green fly ash-based geopolymer concrete via a deep neural network (DNN) and RestNet. The 335 mixes were conducted to obtain the data for the model. Marani et al. [38] employed ML approaches for the prediction of the compressive strength of phase-change materials integrated into cementitious composites, in which the model also gained superior accuracy in terms of prediction. Gupta et al. [39] also present the performance of the ANN approach to predict the mechanical properties of reprised concrete exposed to elevated temperatures, and they also evaluated the impact of input parameters used to predict the performance of the ANN model. Sevim et al. [40] put forward the idea for prediction of cementous composites with waste material applying ML techniques. They use ANN and an adaptive network-based fuzzy inference system (ANFIS) in the study. Ahmad et al. [41] compared the performance of individual and ensemble ML approaches for the compressive strength of fly ash-based concrete, in which they present that ensemble techniques give a better response when R^2^ equals 0.91 as opposed to individual approaches. The main objective of this study is to incorporate the waste material (ceramic waste powder) in concrete to replace the cement by 10 and 20% to analyze the compressive strength and for comparison with that of normal concrete. The novelty of this study is not only relying solely on the experimental results, but also the application of (ML) approaches, ANN, and DT to forecast the compressive strength of ceramic powder-based concrete. The comparison was also made among the actual results obtained from the experimental work at the laboratory and the results from the ML algorithms, for better understanding. Statistical checks and the k-fold cross validation were applied to check the accuracy level between the actual and predicted outcomes. The ANN showed high accuracy with less variance as compared to DT when opting for the available data. The findings of this study are anticipated to promote knowledge and guidance on utilizing CWP as a waste material in concrete to reduce its negative impact on the environment and help to produce the environmentally friendly concrete. In addition, it is also important to investigate the supervised ML approaches for the prediction of the strength property in advance for comparison with the actual result, which would help researchers to investigate the outcomes from the input parameters without consuming time in the practical work.

## 2. Experimental Program and Data Description

This research consists of two phases. Phase one includes the laboratory tests on the material used in order to determine the compressive strength of concrete containing CWP. Phase 2 concerns the application of DT and ANN approaches for the prediction of the compressive strength of CWP-based concrete. Spyder (python 3.8) from the anaconda navigator software was used to run the models using python coding from the sklearn (Scikit-learn) library.

### 2.1. Phase One

In this phase, the properties of the materials used in the experimental program were evaluated, and the compressive strength of normal concrete as well as that containing CWP at certain percentages (10 and 20%) were observed at the age of 28 days. The effect of CWP on concrete mixtures was investigated by performing various tests to determine both fresh and hardened properties of concrete. The workability of fresh concrete was measured by a standard slump test as per ASTM C143 [42]. The hardened property of concrete (compressive strength) developed with age (28 days) and dry density of the specimens were measured.

### 2.2. Phase Two

This phase focused on ANN and DT-based modeling for forecasting the compressive strength of concrete developed during the experimental work at the laboratory. The CS was predicted via ANN and DT models and the accuracy level was compared between the actual and predicted outcomes through coefficient correlation (R^2^) values. The model performance was also investigated through statistical checks as well as other metrics and the k-fold cross-validation method. The error distribution was also evaluated in this phase to confirm the accuracy level of the model.

## 3. Materials

The mixtures of concrete were prepared using ordinary Portland cement (OPC, with a brand name Bestway cement, Islamabad, Pakistan) as a principal binder. The OPC gives confirmation to the ASTM C150 [43]. Type I Cement’s surface area was 385 m^2^/kg. Chemical compositions of the used cement and CWP are presented in Table 1. The CWP used in this research was obtained from the local tile industry in Gujrat city of Pakistan. Initially, the available ceramic waste was wet. The moisture content at that stage was 29% by mass. After this, the CWP was allowed to dry, and it was observed that the largest-size particles are available, and after being sieved, it was also reported that the amount passing the 300 μm sieve was a limited amount. Therefore, the CWP was ground using a wooden-type grinder (locally prepared). The specific surface area (SSA) of the CWP was then measured by air-permeability (Blaine test, MATEST, Treviolo, Italy) and was 562 m^2^/kg.

**Figure 1:**
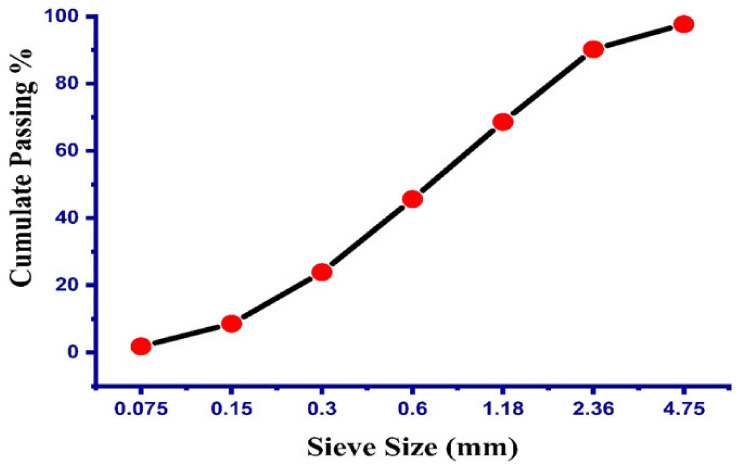
Grain size curve of fine aggregate.

**Figure 2:**
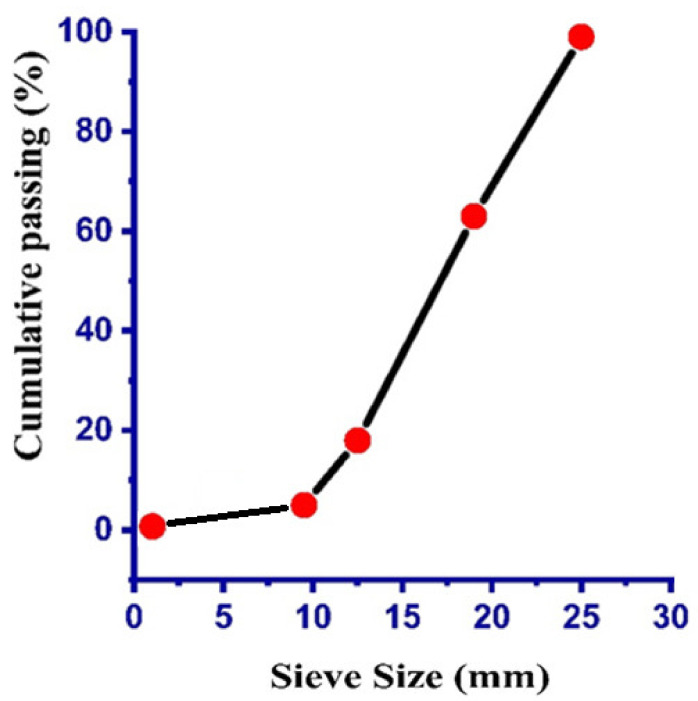
Grain size curve for coarse aggregate.

The coarse aggregate employed in this experimental program was natural crushed stone (igneous rock, Margalla Hills, Pakistan) with a nominal size of 10 and 20.5 mm. The specific gravity for the coarse aggregate was noted as 2650 kg/m^3^ and the water absorption was 1.2%. The grading analysis for the fine aggregate (Margalla Hills, Pakistan) and coarse aggregate are listed in Table 2 and Table 3, respectively, while the gradation curve for fine and coarse aggregates can be seen in Figure 1 and Figure 2, respectively. The specific gravity of the fine aggregate was 2670 kg/m^3^ and water absorption was noted as 2.2%. The physical properties of the cement used are illustrated in Table 4. In addition, the view of the experimental work at different stages can be seen in Figure 2.

The materials in this study have been used for two different processes, for the experimental work performed in the laboratory and as an input parameter used to run the model for the predicted outcome. The data in the arranged form used to run the model can be seen in annexure A. Python coding was used via the anaconda software to run the model. The six parameters (cement, ceramic waste powder, water, fine aggregate, coarse aggregate and age) were used as inputs and one parameter (compressive strength) was used as the output for the ANN model. The descriptive analysis with the mathematical indication of the variables used to run the models, along with their ranges, are listed in Table 5, while the spearman rank correlation coefficients for the parameters are shown in Table 6.

### 3.1. Mix Proportions and Mix Designs

A total of 60 mix designs were prepared with different water cement ratios, from which 20 mix designs were based on the control mixes. A total of two minutes were given for the mixing of plain concrete in the concrete mixing machine, while three minutes were given for concrete containing ceramic waste. A replacement of ordinary Portland cement was carried out by 10 and 20% of ceramic waste powder, from which the 20 mix designs were prepared for each percentage. Six cubes were cast from each batch of mix design. The details of all the mix designs are listed in Appendix A.

### 3.2. Test Methods

The compressive strength of all the concrete samples (cubes) prepared with and without waste material were tested after 28 days of curing. These specimens were tested according to the standard of ASTMC129. The average value of three specimens were taken as the compressive strength of the said mix. Moreover, fresh properties like slump tests were also carried out for each mix as per the ASTM standard.

### 3.3. Artificial Neural Network (ANN)

**Figure 3:**
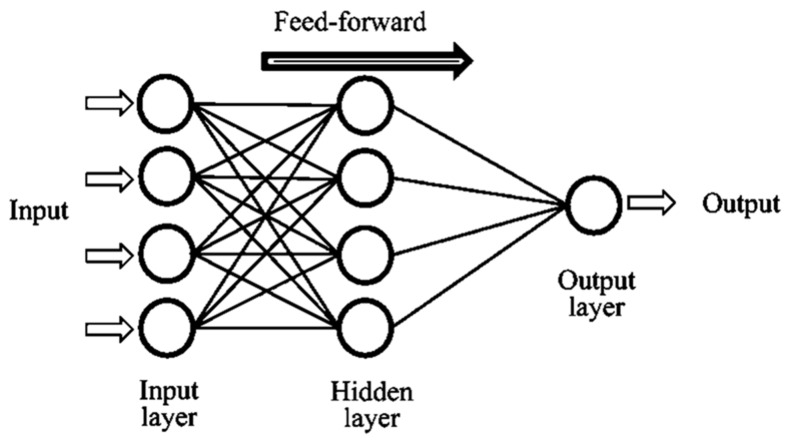
The function of the artificial neural network (ANN) model.

Artificial intelligence (AI) with innovative advances gives a clear indication that artificial neural networks (ANNs) learn to solve multiplex problems in a limited time [44]. ANNs are the tools of non-linear statistical data modeling connections among the input and output data, which may be an adaptive system that can alter its structure based on the details that proceed via the network during the process of learning. The neurons are arranged in layers of feed-forward networks. In the different layers, all available neurons are attached to one another, though in the same layer, no attachment is found between the neurons. Usually, the initial layer is called the input layer, which indicates the input parameters of the ANN, and an equal number of neurons in the input results in the ANN having an equal number of neurons as the problem output. The hidden layers are located between these two layers. The equal number of hidden layers and the equal number of neurons in every layer may not be recognized beforehand, which is because of the problem under exploration [45,46,47]. The ANN opted for a loop from the from the input to the output of the hidden layer. The process of the ANN model can be seen in Figure 3. The activation function used in this study was adopted from previously published articles [48].

## 4. Results and Discussions

### 4.1. Slump Tests

**Figure 4:**
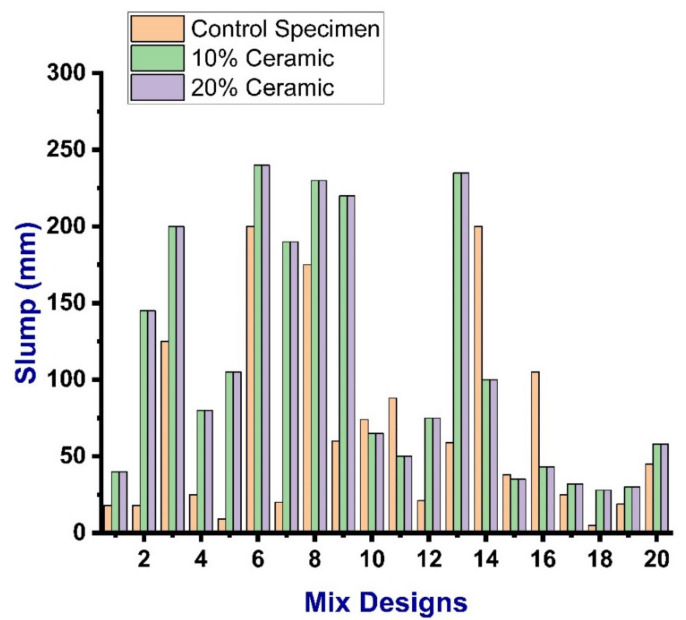
Slump values of the mixes.

The slump tests were carried out for all mixes at a room temperature of 25 °C ± 1 °C as per ASTM C143 [49]. The slump test was caried out in such a way that the clean slump cone was initially placed on the horizontal nonporous and smooth base plate. The cone was then filled with fresh concrete in four equal layers, in which tampering was done by applying 25 strokes to each layer by assuring that the compaction was uniform. The surface of the filled cone was then leveled with the trowel. After that, the mold was resigned from the concrete immediately, yet slowly, in the upward direction. The slump values were then measured by the difference between the height of the mold and the height point of the concrete sample being tested. It was observed that the workability of the control mix varied in all mixes with a maximum slump value of 200 mm and a minimum value of 5 mm. In comparison, the slump test value for the replacement of 10% ceramic waste and 20% waste was reported to be almost similar, as illustrated in Figure 4.

### 4.2. Dry Density of Specimens

**Figure 5:**
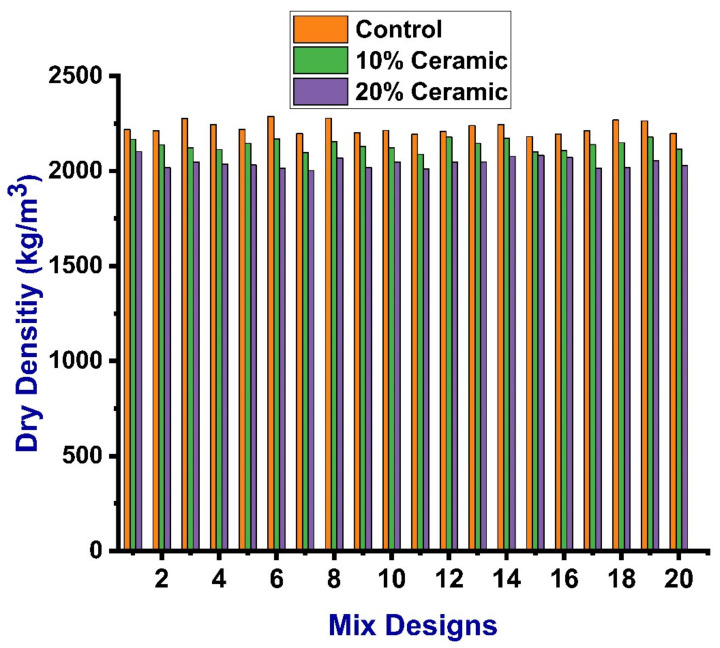
Indicating dry densities of all specimen of each mix.

The densities of the specimens were also evaluated in kg/m^3^ as per ASTM C138 [49]. The density of the specimens was calculated at a room temperature of 25 °C ± 1 °C. The mass of the specimen was calculated using a digital weight balance (MATEST, Treviolo, Italy) and the dimensions were measured with a measuring tape in order to be used in the formula of mass per unit volume to calculate the density of the specimen. The results of the densities were close to each other for all mixes with less margin. The maximum and minimum values for the control mix were 2228 and 2181 kg/m^3^, respectively. Specimens with 10% ceramic waste gave the maximum and minimum values for dry densities of 2178 and 2002 kg/m^3^; similarly, these values for the specimen with 20% ceramic waste were 2103 and 2002 kg/m^3^, respectively, as depicted in Figure 5.

### 4.3. Compression Test

**Figure 6:**
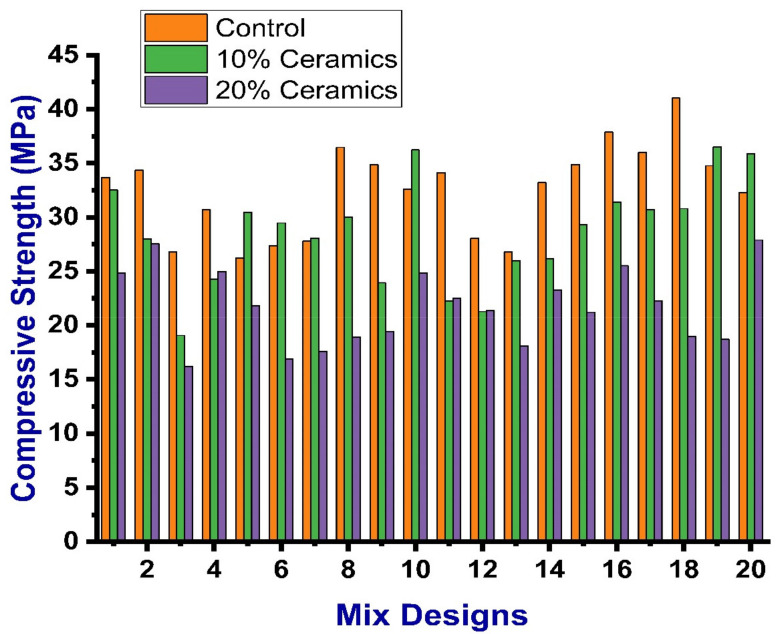
Compressive strength of the specimens of various mixes.

The compressive strength test for all concrete specimens was conducted in compliance with the guidelines of ASTM C39 [50]. The compression testing (on cubes) was done on the compression testing machine (tecnotest, Treviolo, Italy) with the capacity of 2000 kN at a room temperature of 25 °C ± 1 °C. For the prevention of stress concentration, smooth wooden planks with a thickness of about 3 mm were placed at each end of the specimen connected to the loading plate of the compression testing machine. The maximum, minimum, and average values of the compressive strength for the control mix were 41.03, 26.23, and 32.48 MPa, respectively. The maximum, minimum, and average values of the compressive strength containing 10% CWP were 36.5, 19.07, and 28.61 MPa, respectively. Similarly, these values for 20% CWP were reported as 27.9, 16.2, and 21.63 MPa. The results of compressive strength for each mix can be seen in Figure 6. For the compressive strength of all mixes, the average values of the specimens were taken as the test result. If the difference among the minimum and maximum values of the three results was up to 15% of the mean values, then the obtained mean value was selected as the compressive strength result. If any of the results showed more than the 15% of the median value, then the data of the test were considered as invalid, which ultimately resulted in repetition of the mix. However, no mix repetition was done for the mixes where the difference between the minimum and maximum value was not more than 15% of the mean value.

## 5. Artificial Neural Network (ANN) and Decision Tree (DT) Model Results and Analysis

### 5.1. Statistical Analysis

**Figure 7:**
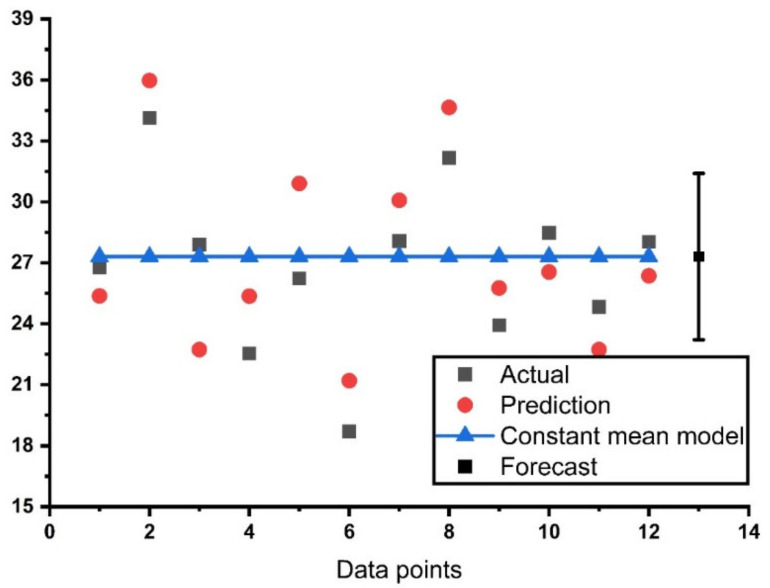
Distribution of constant mean model result.

**Figure 8:**
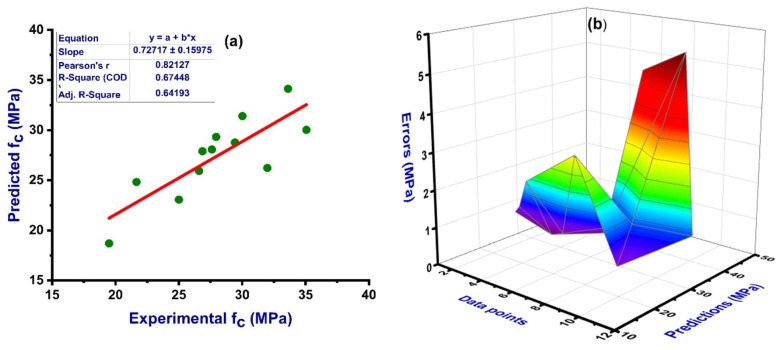

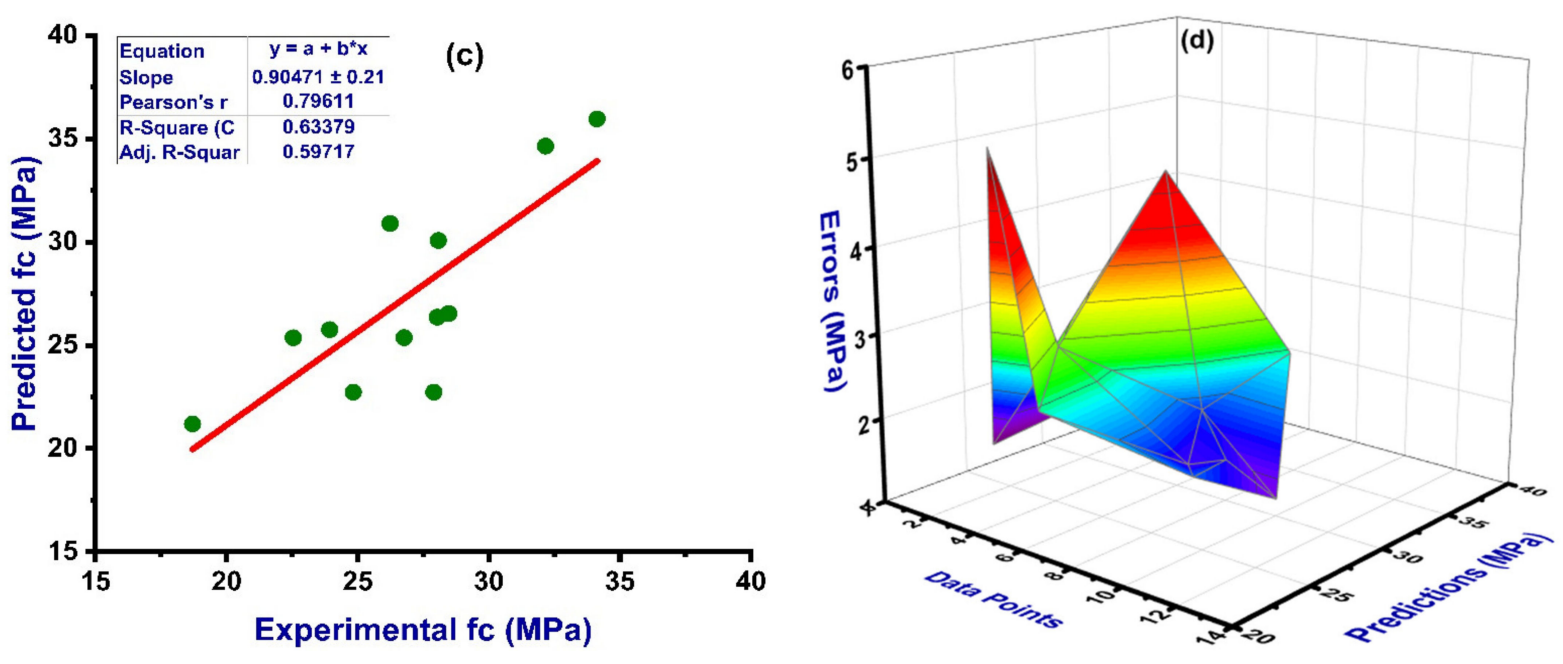
Result of the numerical analysis representing the relation between the actual and predicted variable as well as the distribution of the errors for ANN model (**a**,**b**); DT (**c**,**d**).

The general distribution of the actual, predicted, and constant mean results of the model can be seen in the Figure 7. The same trend was adopted in the study to evaluate the constant mean model. The statistical analysis result for the actual compressive strength and the outcome from the artificial neural network (ANN) and decision tree (DT) models along with their error distribution is presented in Figure 8 The ANN model shows a strong relation indicated from the correlation coefficient value of R^2^ equal to 0.67 between the actual and predicted output, as well as with less variance, as presented in Figure 8a, while its error distribution can be seen in Figure 8b. The error distribution in Figure 8b presents information about the average error of the training set, which is equal to 1.90 MPa. However, the maximum and minimum values of the error were noted as 5.75 and 0.46 MPa, respectively. In addition, 41% of the data showed error between 0.45 and 1.0 MPa and 25% of the error data lies above 2.0 MPa as shown in Figure 8b. Meanwhile, the relation between the actual and predicted outcome for DT model can be seen in Figure 8c, which indicates an R^2^ value equal to 0.63, and the error distribution for the DT model is presented in Figure 8d. The distribution of the errors shows an average value equal to 2.53 MPa. However, the maximum and minimum values of the error distribution were 5.16 and 1.39 MPa, respectively.

### 5.2. K-Fold Cross Validation Method

An analysis was done to check the actual performance of the model through the k-fold cross validation method. This method is normally adopted to evaluate the execution level of the employed models. K-fold cross validation involves splitting the randomly set data into k-groups. In this study, 10 groups were prepared from the data and out of these ten, nine groups were utilized for training purposes, and one was employed for the validation of the model. This process was rerun ten times to obtain the average of these iterations. There is a possibility of observing high performance of the model with the application of the k-fold cross validation method. In addition, the employment of the statistical check provides the response of the model towards the prediction as shown in the form of Equations (1)–(5).

(1) $$\mathrm{RMSE}=\sqrt{\frac{\sum_{i=1}^{n}\;{\left(ex_{i}-mo_{i}\right)}^{2}}{n}}$$

(2) $$\mathrm{MAE}=\frac{\sum_{i=1}^{n}\left|ex_{i}\right.-\left.mo_{i}\right|}{n}$$

(3) $$\mathrm{RSE}=\frac{\sum_{i=1}^{n}{\left(mo_{i}-ex_{i}\right)}^{2}}{\sum_{i=1}^{n}{\left(\bar{ex}-ex_{i}\right)}^{2}}$$

(4) $$\mathrm{RRMSE}=\frac{1}{e}\sqrt{\frac{\sum_{i=1}^{n}{\left(ex_{i}-mo_{i}\right)}^{2}}{n}}$$

(5) $$R^{2}=\frac{\sum_{i=1}^{n}\left(ex_{i}-{\bar{ex}}_{i}\right)\left(mo_{i}-{\bar{mo}}_{i}\right)}{\sqrt{\sum_{i=1}^{n}{\left(ex_{i}-{\bar{ex}}_{i}\right)}^{2}\sum_{i=1}^{n}{\left(mo_{i}-{\bar{mo}}_{i}\right)}^{2}}}$$

where, $ex_{i}$ = experimental value, 

$mo_{i}$ = predicted value, 

${\bar{ex}}_{i}$ = mean experimental value, 

${\bar{mo}}_{i}$ = mean predicted value obtained by the model, 

*n* = number of samples.

**Figure 9:**
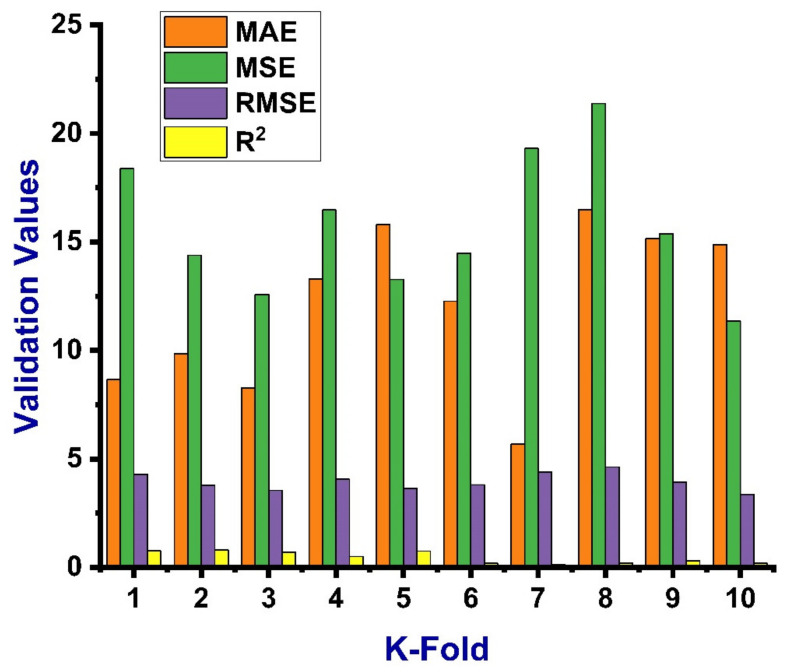
Statistical Indication for k-fold cross validation for the ANN model.

**Figure 10:**
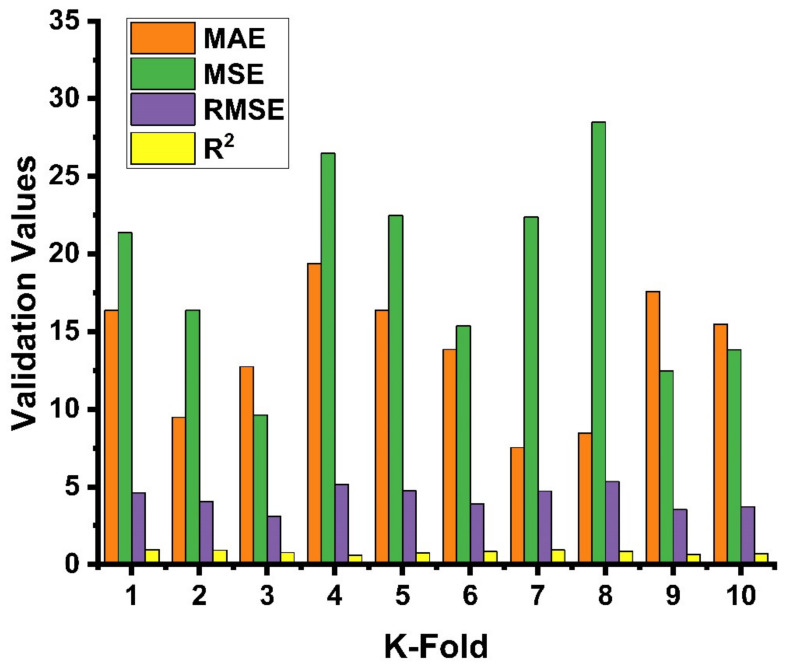
Statistical Indication for k-fold cross validation for the DT model.

The parameters correlation coefficient (R^2^), mean square error (MSE), mean absolute error (MAE), and root mean square error (RMSE) were applied to assess the obtained result of the cross-validation for both ANN and DT as shown in Figure 9 and Figure 10, respectively. The ANN model gives the reflection of less errors and a better R^2^ value, equal to 0.67, indicating high accuracy of the predicted result, while DT gives the R^2^ value of 0.63 and seems close to the ANN result with a smaller margin. The average value of the correlation coefficient (R^2^) for the ANN model was equal to 0.44 with the maximum and minimum values of 0.80 and 0.12, respectively. The result of the MAE was also reported with average, maximum, and minimum values of 12.03, 16.49, and 5.67 MPa, respectively. These values of the same order for MSE were 15.69, 21.37, and 11.34 MPa. Similarly, the RMSE gives maximum and minimum values of 4.62 and 3.37 MPa, respectively, as depicted in Figure 9. The average vale of R^2^ for DT was 0.10, with maximum and minimum values equal to 0.96 and 0.60, respectively. While the average, maximum, and minimum values of the MAE were 19.37, 7.53, and 3.33 MPa, these values for MSE were 28.48, 9.63, and 5.35 MPa, respectively, as shown in Figure 10. Moreover, the data from k-fold cross validation of the ANN model and the information of the statistical checks can be seen in Table 5 and Table 6, respectively. Statistically, the MAE gives a value of 6.94 MPa, the MSE value was 20.76, and RMSE shows its result to be equal to 4.55, as illustrated in Table 6.

This research describes the application of ceramic waste powder in concrete at 10 and 20% replacement of the ordinary Portland cement (OPC) and analyzes the effect on the compressive strength (CS) of concrete and the prediction of CS through the ANN model. The supervised machine learning algorithm (ANN) model outcome for CS was compared with the actual result. The replacement of OPC with ceramic waste in concrete affects the strength properties with less variance and was utilized successfully with appreciable results. In addition, the ANN model also shows an impressive result in terms of forecasting the compressive strength of the concrete containing ceramic waste. The indication of a better response of the ANN model can be visualized from the coefficient correlation (R^2^) value, which was equal to 0.67 in this study, indicating the strong relation between the actual and predicted outcomes, as depicted in Figure 8a. The sklearn (Scikit-learn) library was used which normally takes 80% of the data for training purposes and 20% for testing. The values of the statistical checks (MAE, MSE, and RMSE) are illustrated in the Table 7. In addition, the actual and predicted values of the ANN and DT models can be seen in the Appendix B.

## 6. Conclusions

This study explains the behavior of concrete’s compressive strength when ceramic waste is used as a partial replacement of ordinary Portland cement at certain percentages (10 and 20%), as well as the predicted outcome of the CS from the ANN model. The results from the experimental work, compared with the outcomes from the supervised machine learning algorithm (ANN), shows innovation for research work. The ANN model shows a strong relation through the linear correlation coefficient (R^2^) and low values of the parameters describing the errors of the forecasting. The following conclusions can be drawn from the study.

- Ceramic waste powder is very effective for replacing the OPC at certain percentages reducing the environmental risks (e.g., land pollution).
- This waste can be utilized in concrete where the normal strength of concrete is recommended.
- ANN and DT are very useful supervised machine learning (ML) approached for the prediction of compressive strength of any type of concrete and can be favorably used for this purpose.
- In comparison, the ANN model shows better accuracy with less variance between the actual and predicted result by indication of the R^2^ value equal to 0.67, as opposed to DT, which gives a value of R^2^ equal to 0.63.
- The K-fold cross validation method also proved the high performance of the ANN model.

The two positive aspects of this research are the utilization of waste material in concrete minimizing the environmental risks, and the other is the prediction of CS at an initially high accuracy. To obtain a desired CS, it normally takes 28 days by employing the hit and trial method, which is a time-consuming task. ML algorithms can be successfully used without investing money and consuming significant time. However, more research is still required for the evaluation of the best-performing ML approach.

- Other various types of ceramic waste (white paste for twice-fired ceramic and white paste for sanitary ware) can be used in concrete as a partial replacement of OPC to check the other mechanical properties of concrete (like flexural and split tensile strength).
- The data points can be increased with the practical work to obtain a better response from the models.
- More checks and analysis can be applied to evaluate the model’s performance (like sensitivity analysis).

## Figures and Tables

**Table 1:** Chemical compositions of cement and CWP (all presented values in %).

Ingredients	Cement (%)	Ceramic Waste Powder (CWP) (%)
Silicon dioxide (SiO_2_)	19	66.50
Aluminum oxide (Al_2_O_3_)	9.87	19.50
Ferric oxide (Fe_2_O_3_)	3.46	0.82
Magnesium oxide (MgO)	1.63	2.40
Calcium oxide (CaO)	60	1.85
Sodium oxide (Na_2_O)	0.84	-
Potassium oxide (K_2_O)	1.19	-
Phosphorus pentoxide (P_2_O_5_)	0.063	-
Sulfur trioxide (SO_3_)	2.63	0.10
Others	1.317	8.83

**Table 2:** Grading analysis of fine aggregate.

Sieve Diameter	Retained Weight (g)	% Retained	Cumulative % Retained	Cumulative % Passing
4.75 mm	23.38	2.34	2.34	97.66
2.63 mm	74.5	7.45	9.79	90.21
1.18 mm	216.14	21.61	31.4	68.6
600 μm	229.95	23	54.40	45.6
300 μm	217.2	21.72	76.12	23.88
150 μm	153.12	15.31	91.43	8.57
200 μm	67.78	6.78	98.22	1.79
<200 μm	15.4	1.54	99.75	0

**Table 3:** Grading analysis of coarse aggregate.

Sieve Diameter	Retained Weight (g)	% Retained	Cumulative % Retained	Cumulative % Passing
25.4 mm	0	0	0	100
19 mm	1093	36.43	36.43	63.57
12.7 mm	1327	44.23	80.67	19.33
9.5 mm	462	15.4	96.07	3.93
<9.5 mm	104	3.47	99.53	0

**Table 4:** Physical properties of cement.

Properties	Values
Specific density (kg/m^3^)	3050
Average particle size (µm)	20
Blain fineness (cm^2^/g)	1720
Initial setting time (min)	95
Final setting time (min)	360
Loss on ignition (%)	1.03
Blain fineness (cm^2^/g)	1720

**Table 5:** Descriptive analysis of the parameters.

Parameters Description	Cement	Waste	Fine Aggregate	Coarse Aggregate	Water
Mean	13.49705	1.49965	18.19705	35.3611	6.3862
Standard Error	0.340103039	0.165085	0.665502	1.14417	0.15477
Median	13.2365	1.4705	18.096	32.834	6.495
Standard Deviation	2.634426811	1.278746	5.154953	8.862703	1.198846
Sample Variance	6.940204625	1.63519	26.57354	78.5475	1.437232
Kurtosis	0.041478386	−1.17781	2.125386	−0.94989	0.006432
Skewness	0.458010591	0.205647	−0.45923	0.267513	0.007548
Range	12.738	4.169	26.211	32.107	5.088
Minimum	8.109	0	3.808	19.392	3.852
Maximum	20.847	4.169	30.019	51.499	8.94
Sum	809.823	89.979	1091.823	2121.666	383.172
Count	60	60	60	60	60

**Table 6:** Spearman rank correlation coefficients for the parameters in this study.

Parameter	Cement	Waste	Fine Aggregate	Coarse Aggregate	Water	Compressive Strength (CS)
Cement	1	-	-	-	-	-
Waste	−0.28861988	1	-	-	-	-
FA	0.672262724	0.153871621	1	-	-	-
CA	0.50502207	0.115597808	0.752079311	1	-	-
Water	0.735129108	0.168261584	0.616811018	0.530847489	1	-
CS	0.274814801	−0.721281675	0.014898041	−0.079462241	−0.255920482	1

**Table 7:** Statistical check.

Machine Learning Algorithm	MAE (MPa)	MSE (MPa)	RMSE (MPa)
Artificial neural network (ANN) model	6.94	20.76	4.55
Decision Tree (DT)	6.12	17.98	4.29

## Data Availability

The data presented in this article are available within the article.

## Appendix A

**Table A1:** Details of all mix designs.

Cement (kg/m^3^)	Ceramic Waste Powder (kg/m^3^)	Fine Aggregate (kg/m^3^)	Coarse Aggregate (kg/m^3^)	Water (kg/m^3^)	Compressive Strength (MPa)
528.03	0.00	497.17	1302.63	200.64	33.67
665.95	0.00	250.19	1274.08	253.08	34.35
464.68	0.00	531.29	1151.07	271.22	26.77
572.54	0.00	588.52	1071.99	228.61	30.70
512.83	0.00	538.46	1123.09	225.17	26.23
579.32	0.00	550.36	973.26	274.03	27.37
499.92	0.00	569.90	1174.81	219.86	27.78
562.88	0.00	596.64	1035.73	247.60	36.47
480.45	0.00	590.95	1205.96	221.00	34.87
579.76	0.00	678.29	997.14	222.45	32.60
541.48	0.00	682.25	990.92	228.20	34.13
449.15	0.00	543.46	1311.50	178.27	28.03
433.85	0.00	620.42	1223.50	191.40	26.80
482.04	0.00	636.27	1137.59	198.24	33.20
423.45	0.00	571.66	1350.80	186.32	34.87
501.72	0.00	717.43	1013.44	196.79	37.87
481.51	0.00	693.36	1136.37	185.24	36.00
389.09	0.00	579.74	1330.67	188.51	41.03
413.98	0.00	637.55	1237.83	174.54	34.77
476.71	0.00	700.77	1148.90	172.92	32.27
465.11	51.67	486.58	1274.90	196.37	32.53
598.29	66.51	249.76	1271.88	252.64	28.00
403.15	44.78	512.13	1109.57	261.45	19.07
507.42	56.37	579.52	1055.60	225.11	24.27
479.21	53.26	559.08	1166.08	233.79	30.47
538.41	59.82	568.32	1005.03	282.97	29.47
448.20	49.80	567.72	1170.31	219.02	28.07
501.79	55.74	590.97	1025.87	245.25	30.00
439.91	48.89	601.19	1226.84	224.83	23.93
514.63	57.18	669.03	983.53	219.41	36.23
482.85	53.63	675.96	981.79	226.10	22.23
410.09	45.57	551.33	1330.51	180.85	21.27
393.22	43.68	624.78	1232.10	192.74	25.97
441.65	49.06	647.76	1158.13	201.82	26.17
383.85	42.66	575.79	1360.56	187.67	29.33
456.18	50.69	724.79	1023.82	198.80	31.40
439.97	48.89	703.94	1153.71	188.07	30.70
359.86	39.97	595.74	1367.41	193.72	30.80
380.86	42.32	651.69	1265.28	178.41	36.50
425.23	47.26	694.51	1138.63	171.37	35.87
405.11	101.27	476.82	1249.32	192.43	24.83
521.69	130.41	244.99	1247.59	247.82	27.55
370.44	92.63	529.45	1147.09	270.28	16.20
451.01	112.77	579.51	1055.60	225.11	24.97
416.23	104.04	546.28	1139.39	228.44	21.80
452.26	113.07	537.06	949.75	267.41	16.87
390.47	97.62	556.41	1147.00	214.66	17.60
429.05	107.27	568.50	986.86	235.92	18.93
372.36	93.08	572.48	1168.26	214.09	19.43
455.34	113.83	665.94	978.98	218.40	24.83
426.03	106.52	671.01	974.59	224.44	22.50
346.05	86.51	523.40	1263.10	171.69	21.37
329.68	82.43	589.32	1162.18	181.81	18.10
388.33	97.09	640.73	1145.58	199.63	23.27
331.04	82.78	558.65	1320.06	182.08	21.20
408.27	102.07	729.75	1030.83	200.17	25.50
378.78	94.69	681.80	1117.43	182.15	22.27
309.47	77.38	576.40	1323.02	187.43	18.97
331.27	82.80	637.70	1238.12	174.58	18.70
372.28	93.06	684.06	1121.49	168.79	27.90

## Appendix B

**Table A2:** Actual and predicted values of the ANN and DT models.

Artificial Neural Network (ANN)		Decision Tree (DT)	
Predictions	Test	Predictions	Test
29.4307	28.77	25.374	26.77
26.8661	27.9	35.9683	34.13
25.0034	23.07	22.735	27.9
27.6048	28.07	25.364	22.54
26.5993	25.93	30.9033	26.23
33.6147	34.13	21.2	18.7
21.6579	24.83	30.0762	28.07
35.0682	30.03	34.648	32.163
27.9408	29.33	25.764	23.93
19.4944	18.7	26.55	28.47
31.9883	26.23	22.735	24.83
30.0159	31.4	26.364	28.03
